# The Recovery Umbrella in the World of Elite Sport: Do Not Forget the Coaching and Performance Staff

**DOI:** 10.3390/sports9120169

**Published:** 2021-12-17

**Authors:** Julio Calleja-González, Stephen P. Bird, Thomas Huyghe, Igor Jukic, Francesco Cuzzolin, Francesco Cos, Diego Marqués-Jiménez, Luka Milanovic, Jaime Sampaio, Isaac López-Laval, Sergej M. Ostojic, Margaret T. Jones, Pedro E. Alcaraz, Xavi Schelling, Anne Delextrat, Meeta Singh, Jonatan Charest, Tomás T. Freitas, Lorena Torres Ronda, Adam Petway, Daniel Medina, Antonio Tramullas, Juan Mielgo-Ayuso, Nicolas Terrados, Chris McLellan

**Affiliations:** 1 Department of Physical Education and Sports, Faculty of Education and Sport, University of the Basque Country, (UPV/EHU), 01007 Vitoria-Gasteiz, Spain; 2 Strength and Conditioning Society, 00118 Rome, Italy; palcaraz@ucam.edu (P.E.A.); adelextrat@brookes.ac.uk (A.D.); 3 School of Health and Wellbeing, University of Southern Queensland, Ipswich, QLD 4305, Australia; stephen.bird@usq.edu.au (S.P.B.); chris.mcmlellan@usq.edu.au (C.M.); 4 Basketball New Zealand, Wellington 6141, New Zealand; 5 UCAM Research Center for High Performance Sport, University of Murcia (UCAM), 30107 Murcia, Spain; thomashuyghe@hotmail.com (T.H.); tfreitas@ucam.edu (T.T.F.); 6 School of Kinesiology, University of Zagreb, 10000 Zagreb, Croatia; ijukic@kif.hr; 7 Technogym SpA, 47521 Cesena, Italy; fcuzzolin@technogym.com; 8 National Institute of Physical Education (INEFC), University of Barcelona, 08038 Barcelona, Spain; cosfrancesc@gmail.com; 9 Faculty of Education, University of Valladolid, 42004 Soria, Spain; diego.marques@uva.es; 10 Faculty of Kinesiology, University of Zagreb, 10110 Zagreb, Croatia; luka.milanovic@kif.hr; 11 Creative Lab Research Community, Research Center in Sports Sciences, Health Sciences and Human Development, CIDESD, University of Trás-os-Montes and Alto Douro, 5001-801 Vila Real, Portugal; ajaime@utad.pt; 12 Department of Physiatry and Nursing, Faculty of Health and Sport Science, University of Zaragoza, 50009 Huesca, Spain; isaac@unizar.es; 13 Faculty of Sport and Physical Education, University of Novi Sad, 21102 Novi Sad, Serbia; sergej@panet.co.yu; 14 School of Kinesiology, George Mason University, Manassas, VA 20110, USA; mjones15@gmu.edu; 15 Institute for Health and Sport, Victoria University, Melbourne, VIC 8001, Australia; xschelling@gmail.com; 16 Department of Sport, Health Sciences and Social Work, Oxford Brookes University, Oxford OX3 0BP, UK; 17 Performance Sleep Medicine, Detroit, MI 48202, USA; meeta@meetasinghmd.com; 18 Department of Psychology, University Laval, Québec City, QC G1V 0A6, Canada; jcharest@centreforsleep.com; 19 Centre for Sleep & Human Performance, Calgary, AB T2X 3V4, Canada; 20 NAR-Nucleus of High Performance in Sport, São Paulo 04753-060, Brazil; 21 Spanish Basketball Federation, 28036 Madrid, Spain; lorenatorres07@yahoo.es; 22 Washington Wizards Athlete Care Department, Washington, DC 20004, USA; adampetway@gmail.com (A.P.); dmedina@monumentalsports.com (D.M.); 23 Aspire Center for Excellence, Doha 29222, Qatar; drtramullas@telefonica.net; 24 Department of Health Sciences, Faculty of Health Sciences, University of Burgos, 09001 Burgos, Spain; juankaya@msn.com; 25 Unidad Regional de Medicina Deportiva, Avilés and Instituto de Investigación Sanitaria del Principado de Asturias (ISPA), 33401 Oviedo, Spain; nterrados@ayto-aviles.es

**Keywords:** recovery, performance, staff, fatigue

## Abstract

In the field of sports science, the recovery umbrella is a trending topic, and even more so in the world of elite sports. This is evidenced by the significant increase in scientific publications during the last 10 years as teams look to find a competitive edge. Recovery is recognized to be an integral component to assist athlete preparation in the restoration of physical and psychological function, and subsequently, performance in elite team sports athletes. However, the importance of recovery in team staff members (sports coaches and performance staff) in elite sports appears to be a forgotten element. Given the unrelenting intense nature of daily tasks and responsibilities of team staff members, the elite sports environment can predispose coaches to increased susceptibility to psycho-socio physiological fatigue burden, and negatively affect health, wellbeing, and performance. Therefore, the aim of this opinion was to (1) develop an educational recovery resource for team staff members, (2) identify organizational task-specific fatigue indicators and barriers to recovery and self-care in team staff members, and (3) present recovery implementation strategies to assist team staff members in meeting their organizational functions. It is essential that we do not forget the coaching and performance staff in the recovery process.

## 1. Introduction

Recovery is a trending topic in the field of sports science [1], and even more so in the world of elite sport. This is evidenced by the significant increase [1,2] in scientific publications during the last 10 years as teams look to find a competitive edge during competitions. Different protocols describe specific recovery methodologies that can be employed in order to achieve more efficient recovery processes, these include (1) recovery strategies [2,3] —foam roll [4,5], massage [6], compression garments [7,8], stretching [9], nutrition [10], active recovery [11], sleep [12], water immersion [13], (2) combinations of recovery strategies [14,15], (3) sport-specific recovery characteristics—soccer [16,17], basketball [18,19], volleyball [20], rugby [21], and combat sports [22], and (4) emerging recovery strategies [23]. Additionally, factors, such as recovery time-periods—post-match [16,17] and during congested schedules [24], have also been investigated, with emerging literature examining recovery specific to female athletes [25] and youth athletes [26,27]. However, it is notable that the recovery of coaching and performance staff in elite sports seems to have been forgotten. This opinion delineates the elite sports organizational structure and specific performance–fatigue characteristics of team staff members and key recovery considerations.

## 2. Recovery Domains

Recovery is a complex, multifactorial issue. Collectively, the scientific literature reports that the primary aim of recovery methods is to accelerate the biological recovery process in shorter time periods [16]. Bird [3] described four essential recovery domains, these being neural, muscular, substrate, and psychological (Table 1). Additionally, self-perception of perceived recovery is also an important consideration, which may affect the balance between happiness and wellness in elite sports athletes [28], especially during periods of congested travel and competition. Key observations from research on recovery in team sports highlight the fatiguing effect of traveling [29,30] in addition to training and competing [31]. As a result, new approaches and practical applications of recovery strategies have been discussed to optimize travel and minimize its negative effects on health and performance in team sports athletes [32], with little mention of coaching and performance staff members [33].

**Figure 1:**
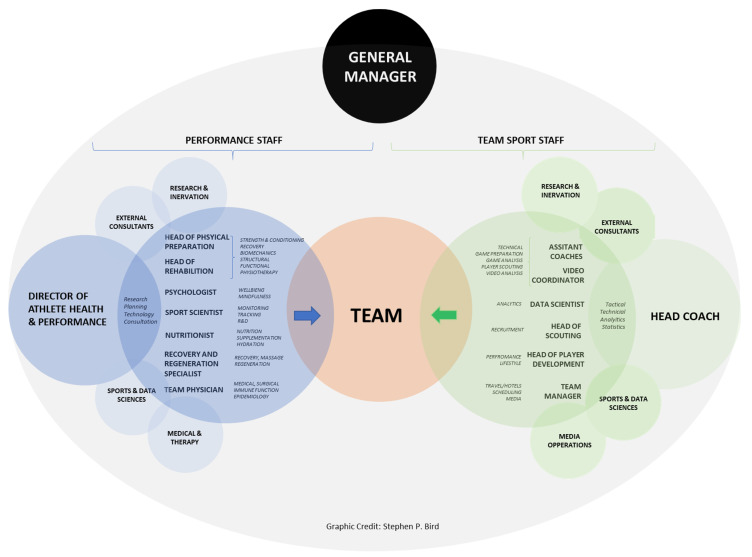
Schematic of professional team organizational structure dynamics comprised of the Team Sport Staff (green shade) and Performance Staff (blue shade) centered around the Team (orange shade). Within the organizational structure, each staff provides expertise related to specific key performance indicators. Modified from Connolly [41].

However, to maximize physical and psychological function and subsequently, performance in elite team sports (National teams, Division I teams), the presence of qualified personnel who are on the front-line helping players, such as the team staff members, is essential [38]. Nowadays, in the lucrative and competitive world of professional team sports, team staff members are made of different profiles [39], which in turn, are organized into two pillars: Team sport coaches and Performance staff. Figure 1 displays an overview of a professional team-sport organizational dynamics structure of team staff members’ roles within elite sports settings. This is a crucial consideration when managing the health and wellbeing of elite coaches and performance staff. This extends the Integrated Performance Health Management and Coaching Model proposed by Dijkstra et al. [40], providing clarity and perspective related to the complex environment of team staff members in elite sports.

## 3. Fatigue in Team Staff Members

There now exists a list of characteristics differentiating expert and competent coaches [42,43], and these attributes can aid in the selection, evaluation, and professional development. Coaches, like athletes, strive to gain employment at an elite level in high-performance sports. However, Kellmann and colleagues [44] highlight that coaching in elite sports is “capricious and dependent upon winning performances and players’ satisfaction” (p. 240). The high-performance sports setting is considered an uncontrollable, unpredictable, and complex environment, with coaches subjected to a multitude of internal and external stressors (i.e., athlete performance, expectations, external scrutiny), that often result in recovery–stress imbalance [44,45]. This may result in coaches presenting with symptoms associated with psycho-socio physiological fatigue burden [46,47,48,49], which can be exacerbated by dehydration, hormonal disturbances, caloric restrictions, or sleep disturbances.

Regarding team staff members (sports coaches and performance staff), common daily tasks and responsibilities, media engagements, study and work commitments, repetitive tasks, over-analysis, thinking about the sport in question, and environmental instability can serve as potential causes of mental fatigue-related issues, with experience and personality as factors of individual susceptibility [50]. Russell and colleagues [50] reported that such issues may lead to more severe consequences, such as disengagement, decreased motivation and enthusiasm, increased displays of emotion and withdrawal, changes in concentration, decreased discipline and attention to detail, as previously described in reports from the elite sporting environment, which can have a negative impact on the team staff performance if left untreated or underappreciated.

In addition, this is an assumption as a lot of team staff members are ex-athletes and remain quite physically active. Additionally, the stress that competition can cause to coaching and performance personnel should be another fundamental factor to consider elevated levels of subjective stress, alpha-amylase activity, and unpleasant emotions suggest that educational programs may be useful for some coaches to manage psychological states during competition [51]. A situation of combined stress for an important championship was found to decrease the level of secretory IgA-mediated immune protection at the mucosal surface, with greater changes observed in the athletes [52]. As such, strategies to minimize the physiological impact are warranted.

## 4. Recovery Considerations for Team Staff Members

The job description of the team staff members can be extensive and demanding [53]. To better cope with the challenges of daily work and with various fatigue sources, support team staff members need to maintain a high level of health, fitness, and mental wellbeing. Fitness, in addition to health protection, plays an important role in mental wellbeing and adaptation to demanding cognitive and social situations [54]. Like athletes, if team staff members possess high levels of health, fitness, and mental wellbeing, they may tolerate fatigue better—that is to say, when in fatigued situations (physical and/or psychological), their capacity to maintain performance will be less affected. Hence, and given the key role of team staff members’ work outputs, and potential impact on the team’s overall performance [44], to that extent, the importance of recovery, especially related to cognitive and psychological fatigue, can contribute to optimizing performance outcomes.

The development of educational recovery resources for team staff members may also help address the identified barriers and improve knowledge [3,55,56]. For example, while team staff members have adequate overall sleep hygiene knowledge [57], some specific areas (e.g., sleep-wake cycle behaviors) warrant further education. Sleep hygiene education is reported to result in positive changes in sleep behavior [34,35]. However, changes in sleep from education in an acute setting may not be sustained following the initial intervention. The progress of instructive sleep resources for team staff members to implement with athletes may help address the identified barriers and improve sleep knowledge. This may be linked to the negative influence of travel, and possible time zone changes [30,32] is another area where education would be beneficial. Empirical findings and practical recommendations are highlighted on sleep, nutrition, recovery, and scheduling strategies to alleviate the negative effects of air travel on health and performance [30,31,58]. Additionally, it would be helpful to have access to a sport psychologist role to address the psychological needs of team staff members to better prepare them for the heavy and indisputable fatigue accumulation during the season [59].

It has been previously demonstrated in athletes that planned disruptions, such as location, competition simulation, punishments and rewards, physical strain, stronger competition, distractions, unfairness or restrictions, can be used to familiarize athletes with pressure, create awareness, develop personal resources, and promote team processes [60]. This is based on the ‘Challenge model of resilience’ presented by Fergus and Zimmerman [61], which suggests that exposure to some adversity can strengthen resistance against future adversity. Consequently, this may also be a consideration to prepare support staff members to deal with pressure under high-stakes circumstances, which they may face frequently. The response to accumulative stress challenges may be exhibited as increased postural tension. Recently, diaphragmatic breathing has been reported to reduce physiological and psychological stress [62]. The potential beneficial effects of diaphragmatic breathing and neural stretching techniques (nerve-directed stretching) [63] may reduce postural tension.

The majority of coaches, regardless of their culture, seem to face difficulties in obtaining professional work and family life (work/family balance), although other coaches indicate that their family life serves as a protection for engagement in a passionate profession [64]. Nevertheless, these potentially conflicting social spheres of work/family balance require special consideration because they have a direct impact on their work performance and potentially on their athletes. Interpersonal relationships within the coaching and the performance teams may also determine the level of stress, fatigue, burnout, and need for recovery. In fact, it is known that quality relationships within a performance department reduce the possibility of injury to athletes [65]. Thus, it is quite certain that the maintenance of a balance between their professional and family commitments and good relations within and between the coaching and performance teams will generate a better mental and physical condition of the staff members of these teams. Strategies such as mindfulness may play a significant role in assisting coaches by strengthening attention and resilience [66]. Birrer and colleagues [36] outline two dimensions of mindfulness that may transfer to work/family balance, these being (1) self-regulation of attention, and (2) the attitude of openness to experience, while a sense of wellness can be promoted through mediation and creative visualization [67]. Furthermore, nonwork outlets are important contributors to fulfill work–life balance [37].

Considering job insecurity as an inherent stressor for coaches, it should be acknowledged and targeted within coaches’ education [68]. Besides moving away from coaching at the elite level, they unanimously mentioned that they changed their approach to coaching to make recovery possible [33]. Therefore, some of the possible means of target recovery that team staff members could use are presented in Table 2. This is an essential consideration given that recovery, as well as social support, might be important in managing stressors associated in the challenging high-performance work environments of full-time coaches. Collectively, this could have a considerable impact on immune health [69].

## 5. Conclusions

The health, wellbeing, and performance of team staff members is of utmost importance in elite sport high-performance organizations, given that their work outputs and capabilities are essential to successful team operation. For these functions to be optimized, recovery strategies targeting the identified fatigue consequences of team staff members should be implemented based on individual fatigue indicators. To assist with the implementation of recovery strategies for coaching and performance staff, integration requires education, individualization, and personalization [3].

Finally, we highlighted that the concepts and practical suggestions related to the recovery of team staff members working in elite sport settings presented in this paper may transfer to staff working within sports schools, sports academies, and/or government sports institutions. Successful implementation will assist team staff members in meeting their organizational functions. Therefore, it is essential that we do not forget the coaching and performance staff in the recovery process.

## Figures and Tables

**Table 1:** Essential recovery domains.

Domain	Recovery Strategy Examples	Evidence	Reference
Neural	Compression, Massage	↑ ↔	[6,7,8]
Muscular	Hydrotherapy, Contrast water therapy, Massage	↑ ↑ ↔	[13,14]
Substrate	Nutrition, Hydration	↑ ↑	[2,10]
Psychological	Sleep, Mindfulness	↑ ↑	[34,35,36]
Sociological	Social interactions (family-friends)	↑	[37]

Level of evidence: Strong = ↑, Inconsistent = ↔.

**Table 2:** Team staff members’ coaching domains, potential fatigue consequences, health risk, and recovery focus areas.

Domain	Title	Primary Role	Game Travel	Potential Fatigue	Health Risk	Recovery Focus	Recovery Strategies	Reference
Performance Coaching	Head Coach	Planning, preparation, coaching	☑	Travel	Sleep disorders	1.Immune health	Immune Health	[69,70]
	Assistant Coach	Logistical support for head coach	☑	Psychological/Emotional	Emotional exhaustion	2.Postural re-sets	Probiotics	
	Head of Player Development	Develop pathway to professional	↔	Asthenopia (eyestrain)	Social isolation	3.Sleep hygiene	Vitamin C	
	Head of Scouting	Athlete identification, recruitment	⊠	Postural fatigue	Back health	4.Mindfulness	Vitamin D	
Performance Health Management	Director, Health, and Performance	Health/performance initiatives	☑/↔	Travel	Sleep disorders	1.Immune health	Postural Re-sets Neural stretching Trigger point therapy Diaphragmatic breathing	[5,62,63]
	Head of Physical Preparation	Athlete physical preparation	☑/↔	Cognitive	Psychological exhaustion	2.Sleep hygiene		
	Head of Rehabilitation	Injury re-conditioning	☑	Emotional		3.Mindfulness		
	Strength and conditioning coach	Physical training of athletes	☑	Psychological				
	Nutritionist	Meal plans, supplements, hydration	↔	Physical				
Sports and Data Sciences	Video coordinator	Create/Edit/log film clips	☑	Cognitive	Eye health (CVS)	1.Postural re-sets	Sleep Hygiene Maintain a regular schedule Minimize electronic use Room temperature (18–21°) Dark (blackout curtains)	[34,35,57]
	Performance analyst	Performance analytics	↔	Asthenopia (eyestrain)	Sleep disorders	2.Sleep hygiene		
	Sport Scientist	Research innervation	↔	Postural fatigue	Carpal tunnel	3.Mindfulness		
	Biostatistician	Statistical analysis	↔		Back health			
Medical and Therapy	Team Physician	Medical diagnosing and treatment	↔	Travel	Psychological exhaustion	1.Sleep hygiene	Mindfulness Relaxation techniques Creative visualization Meditation Desensitization Journaling thoughts	[36,67]
	Athletic Trainer	Athlete taping, injury prevention	☑	Cognitive	Sleep disorders	2.Mindfulness		
	Massage Physiotherapist	Soft tissue treatment	↔	Physical				
	Sport Psychologist	Psychological interventions	↔					
	Osteopath	Musculoskeletal manipulation	⊠					

Abbreviations: Yes = ☑; No = ⊠; Sometimes = ↔; Computer vision syndrome = CVS.

## Data Availability

No data presented in this study.
